# Resolution of the Central Dental Association, Northern New Jersey, on Dr. Atkinson

**Published:** 1891-07

**Authors:** 


### RESOLUTION OF THE CENTRAL DENTAL ASSOCIATION, NORTHERN NEW JERSEY, ON DR. ATKINSON.

WE, the members of The Central Dental Association of Northern New Jersey, having learned with sincere regret of the death of our friend and fellow-member, W. H. Atkinson, M.D., D.D.S., of New York, who, by reason of his great abilities, scholarship, zeal, industry, and self-sacrificing devotion to the interests of the dental profession, and the never-failing willingness to impart his knowledge to all who asked it, he was recognized by us as the most influential member of our profession, a man who devoted his life to its honor and advancement.

During the eleven years of the existence of this Society he has scarcely missed a meeting, and his relations with us have been such, that it is our pleasure and duty to record our high appreciation of him.

That by the death of Dr. Atkinson the dental profession has been deprived of one of its most able and useful members, one whose influence for good will last while dentistry exists.

We have lost one of our best friends, and, as we fondly called him "Father Atkinson," so, indeed, do we feel we have lost our "Father in Dentistry."

We, therefore, extend to his family and to our brother members of the dental profession our sincere sympathy in their great bereavement, and that a copy of these resolutions be sent to the family of the deceased, and that they also be published in the dental journals.

(Signed) J. ALLEN OSMUN,
C. S. STOCKTON,
CHAS. A. MEEKER,
S. C. G. WATKINS,
C. W. F. HOLBROOK,
*Committee.*

